# Coupling Effects of Ionic Surfactants and Electrolytes on the Stability of Bulk Nanobubbles

**DOI:** 10.3390/nano12193450

**Published:** 2022-10-02

**Authors:** Xiaotong Ma, Mingbo Li, Xuefei Xu, Chao Sun

**Affiliations:** Center for Combustion Energy, Key Laboratory for Thermal Science and Power Engineering of Ministry of Education, Department of Energy and Power Engineering, Tsinghua University, Beijing 100084, China

**Keywords:** bulk nanobubble, surfactant, surface charge, surface tension, stability

## Abstract

As interest in the extensive application of bulk nanobubbles increases, it is becoming progressively important to understand the key factors affecting their anomalous stability. The scientific intrigue over nanobubbles originates from the discrepancy between the Epstein–Plesset prediction and experimental observations. Herein, the coupling effects of ionic surfactants and electrolytes on the stability of bulk nanobubbles is studied. Experimental results show that ionic surfactants not only reduce the surface tension but also promote the accumulation of net charges, which facilitate the nucleation and stabilization of bulk nanobubbles. The addition of an electrolyte in a surfactant solution further results in a decrease in the zeta potential and the number concentration of nanobubbles due to the ion shielding effect, essentially colloidal stability. An adsorption model for the coexistence of ionic surfactants and electrolytes in solution, that specifically considers the effect of the adsorption layer thickness within the framework of the modified Poisson–Boltzmann equation, is developed. A quantitative agreement between the predicted and experimental surface tension is found in a wide range of bulk concentrations. The spatial distribution of the surface potential, surfactant ions and counterions in the vicinity of the interface of bulk nanobubbles are described. Our study intrinsically paves a route to investigate the stability of bulk nanobubbles.

## 1. Introduction

Bulk nanobubbles are gaseous nanodomains (less than 1μm in diameter) that are freely suspended in an aqueous solution [1]. Over the last two decades, bulk nanobubbles have shown great potential for broad applications, seeing that they possess peculiar properties, such as an anomalously long lifetime, a high specific surface area, a high mass transfer efficiency and so on. It is generally believed that bulk nanobubbles are of great significance to enhance the flotation efficiency, especially for fine particles separation [2]. In addition, bulk nanobubbles pave a new route to resolve industrial challenges, such as surface cleaning with a low environmental impact [3]. Supplying oxygen in the form of bulk nanobubbles has already been utilized in the field of water treatment [4,5,6], environmental remediation [7] and agriculture [8]. In medicine, nanobubbles exhibit a better ability to penetrate biological tissue than common ultrasound contrast agent (bubbles with a size of 0.5∼10 μm), which has attracted a growing interest for therapeutic purposes [9,10,11]. Notwithstanding widespread applications, bulk nanobubbles remain controversial from a scientific perspective. The long-term stability of bulk nanobubbles stands at odds with the classical prediction that bubbles undergo either dissolution or growth under atmospherical conditions. Based on the widely accepted Epstein–Plesset theory [12], the nanobubbles are expected to disappear in a few milliseconds or less, driven by the tremendous Laplace pressure, which stems from the surface tension of nanobubbles’ curved surface. However, a broad corpus of experiments have reported that bulk nanobubbles could stably exist in pure water or other aqueous solutions for days even weeks [13,14,15]. The debate continues so far about how to perceive and control the peculiar characteristics of bulk nanobubbles. It is imperative to bridge the gap between fundamental understanding and industrialization in the field of bulk nanobubbles, i.e., their stabilization mechanism.

Various hypotheses have been proposed to account for the stability of bulk nanobubbles. It has been reported that the bulk nanobubbles could be stabilized by coatings, which exert a mechanical stress on the interface that acts against the Laplace pressures [16]. Another dynamic equilibrium model propounds that a gas-permeable contamination adsorbed partly on the bubbles’ surface allows for an influx of the gas, which balances the gas outflux from the regions that are not coated by the hydrophobic material [17]. It happens when the total changes of energy and entropy are zero, maintaining somewhat stable. Moreover, some other alternative visions suggest that the stability arises from the abnormal properties of nanodomains, such as ultrahigh gas density [18], surface polarization interaction [15] and unusual surface tension [19,20]. Nevertheless, most of the above explanations could not adduce strong and independent experimental proof as of yet.

Owing to the fact that the bulk nanobubbles carry electrical charges in some kinds of aqueous solutions, researchers have shown an increased interest in the charge stabilization model recently [21,22,23,24,25]. The surface charges on the bubble surface create an electrostatic pressure to prevent the Laplace pressure driven dissolution, so that the individual nanobubbles might enjoy a thermodynamic equilibrium. Furthermore, the surface enrichment of charges improves the colloidal stability of bulk nanobubbles, which means that the repulsive interaction between adjacent nanobubbles can prevent coalescence [26]. Although bulk nanobubbles are characterized by a negative zeta potential, typically −15 to −40 mV in a neutral pH solution, it remains challenging to link the theoretical model with experimental results. The main reasons come from the uncertainty of an ongoing ambiguity over the zeta potential of a gas bubble, such as repeatability, measuring location and the overlap of the electric double layer [27]. Another issue is that there is no consensus on the origin of the charges at the gas–liquid interface. Moreover, little has been known about the surface charging of nanobubbles, e.g., the origin of these charges and how they are adsorbed to the surface [28,29,30,31,32].

Although it is an open question as to whether bulk nanobubbles are stabilized by the surface charging, the composition and physicochemical properties of solutions, including temperature, pressure, pH, ion strength and impurities, play a vital role in the stability of bulk nanobubbles [21,26,33,34]. One tentative, but speculative mechanism is that the amphiphiles in aqueous solution should not be ignored, as they are directly related to the Laplace pressure. Surfactants are typical amphiphilic molecules that are commonly found in daily life and industrial production. According to whether the surfactant molecules can be dissociated in aqueous solution, they are classified into ionic and nonionic ones. Due to the unique properties of ionic surfactants, they are gradually receiving more attention within the field of nanobubbles. Generally, the ionic surfactant dissociates into the hydrophobic tail part with hydrophilic charged head groups, referred surfactant ions and counterions in aqueous solution [35]. The hydrophobic part, structuring with the hydrophilic charged head groups, has a deep affinity with the gas–liquid interface, leading to the enrichment of surfactant ions at the surface. For the case of coated nanobubbles, the surfactant also serves to reduce the surface tension, thus further slowing down the shrinkage of the nanobubbles. Particularly, the surfactant ions, as charge carriers, may exert an electrostatic pressure on the gas–liquid interface to counteract the Laplace pressure as well. Both factors, rooted in ionic surfactant molecules, appear to contribute to the stabilization of nanobubbles. However, trace amounts of impurities in water are difficult to remove completely, even after very rigorous filtration and purification procedures. While one cannot make the water purer, an easier route to test the hypothesis is to make the water dirtier. For example, more surfactants and counterions could be added to evaluate the influence on nanobubbles. To date, the effect of surfactants on micro- and nanobubbles have been reported qualitatively from experimental perspectives [22,36,37,38,39]. While a significant effect of surfactants on the properties of nanobubbles was indeed observed experimentally, a reasonable and quantitative description of how surfactant molecules are adsorbed to the interface remains elusive due to the uncertainty in theoretical calculations. An in-depth understanding of the adsorption behavior of ionic surfactants at the interface will be beneficial to modulate the properties of nanobubbles, such as stability, size distribution, surface potential, etc. In particular, the presence of salt ions in solution, which also act as charge carriers, cannot be ignored and may have an important influence on the stability of bulk nanobubbles together with surfactants. In addition, several applications of bulk nanobubbles, like flotation and drug delivery, occur in systems containing electrolytes and surfactants. The investigation of the adsorption of ionic surfactant and electrolyte at the gas–liquid interface is of fundamental and practical interest.

In this study, the coupling effects of ionic surfactants, especially anionic surfactants, and electrolytes on the nucleation and properties of bulk nanobubbles are explored systematically. We are concerned primarily with a quantitative description of the role of surfactants and electrolytes in nanobubble stabilization with experiments and theory. We adopt a variety of techniques, such as nanoparticle tracking analysis (NTA) techniques, dynamic light scattering (DLS) techniques and laser Doppler velocimetry (LDV) combined with an electrophoresis technique, to characterize the properties of bulk nanobubbles produced by ultrasonic cavitation. Meanwhile, applying a modified Poisson–Boltzmann equation combined with the interaction between ions and surfactants and incorporating the effect of the adsorption layer thickness, we describe the spatial distribution of the surface potential, surfactant ions and counterions in the vicinity of the interface of bulk nanobubbles quantitatively. We predict that the adsorption of charge carriers results in an accumulation of charges and a reduction of surface tension at the air–water interface, which are responsible for the stability of bulk nanobubbles.

## 2. Materials and Methods

### 2.1. Materials

Ultrapure water with a pH of 6.5 at room temperature 25 ${}^{{}^{\circ}}$C and an electrical conductivity of 18.2 MΩ·cm was obtained by a water purification treatment system (Milli-Q, Merck, Darmstadt, Germany) for all experiments. Sodium dodecylsulfate (SDS, ≥99.0%, GC), hexadecyltrimethylammonium bromide (CTAB, ≥99.0%, GC) and sodium chloride (NaCl, 99.5% AR) were purchased from Sigma Aldrich (Taufkirchen, Germany). The ionic surfactants (CTAB and SDS) were dissociated into their hydrophobic parts with the hydrophilic charged head group and the counterions. The SDS dissociated into ${\mathrm{DS}}^{-}$ and ${\mathrm{Na}}^{+}$, while the CTAB dissociated into ${\mathrm{CTA}}^{+}$ and ${\mathrm{Br}}^{-}$ in aqueous solution.

The critical micelle concentration (CMC), one of the most important parameters of a surfactant, indicates the starting concentration to generate and form micelles. In order to make the influence of the surfactant and electrolyte more apparent, a concentration below the CMC was used. The CMC of SDS and CTAB at 25 ${}^{{}^{\circ}}$C is 8.2 mM and 0.92 mM, respectively. The surfactant molecules and electrolytes with varying concentration were dissolved in pure water using a magnetic stirrer for 30 min at 25 ${}^{{}^{\circ}}$C to achieve complete dissolution.

All glassware was immersed in a 10% sodium hydroxide (NaOH, 99.5% AR, Sigma Aldrich, Germany) at least 30 min and then rinsed with pure water to remove the lipid impurities. Subsequently, the glassware was cleaned with ethanol and pure water ultrasonically. Before experiments, the purified water and all aqueous solutions containing surfactant and electrolyte were initially examined for any possible nanoscale entities using nanoparticle tracking analysis techniques.

### 2.2. Formation and Characterization of Nanobubbles

**Figure 1:**
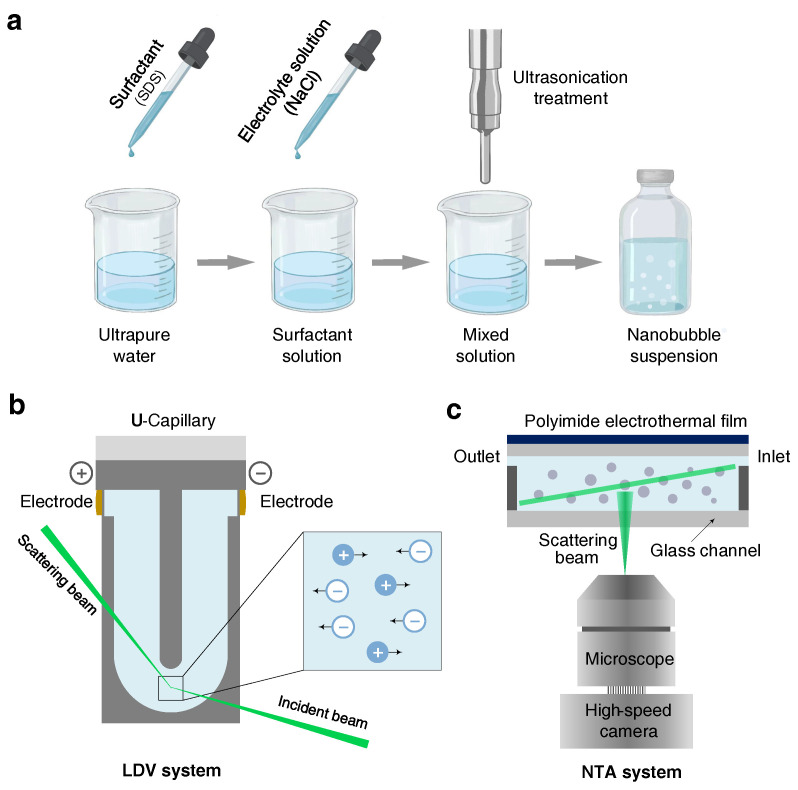
Sketch of bulk nanobubbles generation and characterization. (**a**) Bulk nanobubbles are generated by ultrasonic cavitation method. The surfactant and electrolyte solutions are prepared prior to ultrasonication. (**b**) The nanobubbles are characterized with laser Doppler velocimetry (LDV) combined with an electrophoresis technique and (**c**) nanoparticle tracking analysis (NTA) system.

The ultrasonic cavitation method was adopted to generate the bulk nanobubbles suspensions [14,21,26]. As shown in Figure 1a, 100 mL of a prepared aqueous solution was poured into a clean glass beaker and then treated at 20 kHz using a titanium probe (VCX, Sonics & Materials, Newtown, CT, USA, 750 W) with a varying ultrasonic amplitude and time. Note that it was necessary to clean the probe with ethanol and pure water to eliminate possible contaminants prior to the experiments. During the ultrasonic process, the glass beaker was immersed in a water-recirculating cooler equipment (PolyScience PP15r-40, Niles, IL, USA) in order to maintain the temperature of suspensions at a constant 25 ${}^{{}^{\circ}}$C. To minimize the effects of temperature fluctuations, an intermittent pulse-working mode (ON/OFF: 20/10 s) was adopted. If not specified, the ultrasonic cavitation treatment time was fixed at 15 min. After generation, the bulk nanobubble suspensions were conserved in the airtight glass vials for the following measurements. No other gases were introduced throughout the ultrasonic cavitation and preservation process.

The zeta potential of bulk nanobubbles was measured by a Zetasizer NanoZSE Instrument (ZEN3700, Malvern Instruments, Malvern, UK). The working principle is shown in Figure 1b. When released between electrodes with opposing polarity, charged nanoparticles usually migrate toward the cathode, conforming to the principle of electrophoresis. The nanoparticles’ migration speed at the bottom of the U-shape capillary was obtained by a laser Doppler velocimetry (LDV) method. According to the applied electric field intensity and migration speed, the zeta potential was estimated by the Smoluchowski model [40]. Automatic runs (1∼100) for each sample were performed at least 6 times, and the time interval between each measurement was 10 s. The temperature of all the measurements was set at 25 ${}^{{}^{\circ}}$C.

A nanoparticle tracking analysis (NTA) system built by us was used to determine the size distribution and bubble number concentration of bulk nanobubbles suspensions [21,26]. The NTA system contained a microfluidic sample cell, a laser and a dark-field microscope (IX73, Olympus, Tokyo, Japan) equipped with a long-working-distance objective (×20 magnification), as depicted in Figure 1c. The laser with a wavelength of 520 nm illuminated the nanobubbles suspension in the glass chamber with a thickness of 500 μm. Bulk nanobubbles, acting as the scattered spots, were visualized by a high-sensitivity CCD camera. Nanobubbles in the selected view were counted to acquire the bubbles number concentration within the observing volume. Meanwhile, the Brownian motion of bulk nanobubbles was recorded by the camera at a frame rate of 30 fps. Further, the trajectory of nanobubbles in the video was processed to estimate the diffusion coefficient via ImagesJ/Fiji [41] and NanoTrackJ [42]. Hence, the hydrodynamic diameter was calculated on the Stokes–Einstein equation. To ensure the reliability of measurements, the NTA system was evaluated systematically in our previous work and exhibited good performance in terms of accuracy and resolution.

### 2.3. Measurement of Surface Tension

The surface tension of surfactant solutions was determined by a surface and interface tensiometer (DCAT25, DataPhysics, Filderstadt, Germany), based on the Wilhelmy plate method. The thin plate was made of platinum usually on the order of a few square centimeters in area. The platinum plate was thoroughly cleaned with pure water applying ultrasonication and then burned using an alcohol lamp to remove organic contamination. When the particular plate fixed on the tensiometer was partially immersed in a pool of liquid at a certain speed, a thin film formed. The force on the plate owing to wetting was tested and applied to obtain the surface tension. The uncertainty was about ±0.2 mN/m. The measurements were calibrated with pure water and ethanol (≥99.0%, AR, Titan, Zhuhai, China) at 25 ${}^{{}^{\circ}}$C. The surfactant and electrolyte mixture experienced an equilibrium time, hence multiple measurements were performed for each sample until the surface tension measured became constant. Three measurements were performed at a temperature of 25 ${}^{{}^{\circ}}$C and the average was obtained for each example.

## 3. Results and Discussion

### 3.1. Effect of Surfactant and Electrolyte on Nucleation of Bulk Nanobubbles

**Figure 2:**
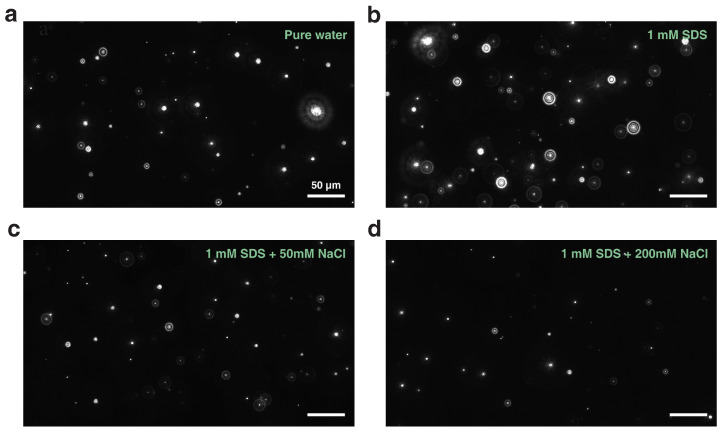
NTA snapshots of bulk nanobubbles generated in (**a**) pure water, (**b**) 1 mM SDS solution, (**c**) 1 mM SDS solution with 50 mM NaCl added and (**d**) 1 mM SDS solution with 200 mM NaCl added.

There have been some fragmentary works exploring the effect of surfactants or salts on the properties of bubbles ranging from the macroscale to the nanoscale [17,37,38,43,44,45]. However, a question naturally arises: would the characteristics of bulk nanobubbles be different if surfactant molecules and salts (with a high enough concentration) coexisted in solution? First, we explore the effect of surfactants and electrolytes on the nucleation of bulk nanobubbles jointly. Note that the observed nanoentities produced by the ultrasonication method have been confirmed to be gaseous nanobubbles experimentally [14,28,46]. Hereby, no more detailed evidence is presented. Moreover, no feasible method for in situ detection of gases inside bulk nanobubbles has been reported so far. In our opinion, we presumed that the main component of the gas inside the nanobubble was a mixture of air and vapor. However, it needed to be confirmed further. In Figure 2, we present four representative snapshots of bulk nanobubbles suspended in aqueous solutions, which were captured by the NTA system. We found that bulk nanobubbles could nucleate in pure water as well as various aqueous solutions containing surfactants and electrolytes, as long as there was enough energy input. Intuitively, there were more nanobubbles present in the solution with anionic surfactant (SDS) than in pure water. Nevertheless, the extra addition of salts (NaCl) to the surfactant solution reduced the concentration of bubbles, and this effect became more pronounced with a higher salt concentration. It was confirmed that the nucleation of bulk nanobubbles indeed depended strongly on the surfactants and electrolytes in aqueous solutions.

**Figure 3:**
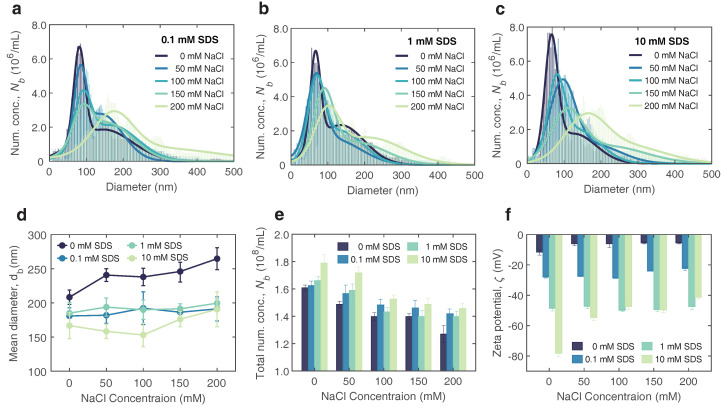
Effect of anionic surfactant (SDS) and electrolyte (NaCl) on bulk nanobubbles. (**a**–**c**) Size distribution of bulk nanobubbles generated at various concentrations of surfactant and electrolyte aqueous solution. (**d**–**f**) Mean bubble diameter $d_{b}$, number concentration $N_{b}$ and zeta potential ζ bulk nanobubbles generated in surfactant and electrolyte solution at varying concentrations. Error bars represent the standard deviation of at least six measurements.

To further investigate the influences mentioned above, we measured the size distribution of bulk nanobubbles generated in aqueous solutions with anionic surfactants and electrolyte coexisting, as shown in Figure 3a–c. Herein, three typical concentrations of SDS and electrolyte concentration ranging from to 0 mM to 200 mM were considered. The results showed that bulk nanobubbles could exist in all cases, with a similar range of size distributions (<∼500 nm in diameter). The size distribution of bulk nanobubbles narrowed slightly with the increasing concentration of SDS from 0.1 mM to 10 mM. Taking Figure 3a as an example, the size distributions of the bulk nanobubbles generated in 0.1 mM SDS solutions with a varying NaCl concentration from 0 mM to 200 mM are plotted. It can be seen that the added NaCl in surfactant solutions affected the nucleation of bulk nanobubbles significantly. With an increasing NaCl concentration, the size distribution of bulk nanobubbles broadened with the main peak shifting towards the right, i.e., a larger diameter. This trend was universal, even for solutions with high concentrations of added surfactants, as depicted in Figure 3b,c. Interestingly, the higher the surfactant concentration, the more pronounced the change in size distribution with the increasing electrolyte concentration.

The mean bubble diameter could be calculated based on the size distribution, which is illustrated in Figure 3d. Starting from the bulk nanobubbles generated in pure water, the mean bubble diameter declined slightly with an increasing SDS concentration, but increased as the NaCl concentration increased, which was consistent with previous experimental results [36,46,47]. For the cases of surfactant and electrolyte coaddition, the nucleation process of bulk nanobubbles was more complex. In Figure 3d, it appears that the mean bubbles size is weakly dependent on NaCl concentration. Based on the charge stabilization model, for individual bubbles in thermodynamic equilibrium, the outward electrostatic pressure may balance the inward Laplace pressure and thus prevent gas diffusion to a certain degree. With an increasing concentration of NaCl in the SDS solution, the surface tension reduced owing to more surfactant adsorption while the surface electrostatic interaction increased due to the enhanced surface charge density. The stable equilibrium size of individual nanobubbles was determined by the interaction of multiple forces at the interface. The adsorption of surfactant ions and salts may break the initial equilibrium state, which may lead to an unpredictable bubble evolution (swell or shrinkage).

The bubble number concentration $N_{b}$ revealed the joint influences of surfactants and electrolytes on bulk nanobubbles as well, as shown in Figure 3e. In the cases of bulk nanobubbles generated in solutions without NaCl, the bubble number concentration rose with the increasing SDS concentration, indicating that the surfactant could facilitate nucleation of nanobubbles. On the contrary, the presence of electrolytes appeared to cause a reduction in bubble concentration. It may be attributed to the enhanced ion shielding effect near the nanobubbles when adding electrolyte into pure water or a surfactant solution, and further bubbles coalescence. The colloidal stability or instability of bulk nanobubbles suspension resulting from the presence of surfactants and electrolytes was confirmed by the experimental results of the zeta potential ζ, as shown in Figure 3f. Since the bulk nanobubbles formed for all cases exhibited a negative zeta potential, hereinafter we discuss the magnitude of the zeta potential regardless of sign. Apparently, the zeta potential increased almost linearly with the concentration of anionic surfactant (SDS), suggesting that the addition of SDS improved the colloidal stability of bulk nanobubbles. The charged surface prevented the coalescence of adjacent bubbles, leading to a more stable bulk nanobubble system. As the concentration of electrolyte dissolved in solutions increased, the zeta potential dropped significantly owing to the enhanced ion shielding effect in the vicinity of the surface of the bulk nanobubbles. The nanobubbles under the Brownian motion were easier to coalescence, which explained the reduction of bubble concentration partly.

Taken together, it could be inferred that the effects of surfactants on bubble nucleation was mainly due to the reduced surface tension and enhanced surface charge density. On the one hand, the reduction of the surface tension of the solution led to a substantial lowering of the nucleation threshold [48], which allowed more bubbles to form under certain ultrasound energy inputs. On the other hand, a high electrostatic repulsive force not only counteracted the Laplace pressure partly but also suppressed coalescence between the neighboring bubbles, which was affirmed by the high zeta potential. However, the addition of electrolyte weakened the electrostatic interaction owing to the ion shielding effect, which resulted in a wider size distribution, larger mean bubble size, lower bubble concentration and zeta potential.

### 3.2. Modeling and Validation of Surfactant Ion Adsorption

The surface charging process plays a vital role in stabilizing bulk nanobubbles, which has been confirmed by numerous researchers [23,49]. Nevertheless, both the gas–liquid interfacial molecular structure and the origin of the positive or negative zeta potential of bulk nanobubbles in aqueous solution are heavily controversial. There are several explanations for the origin of charges at the interface, including the adsorption of ions and surfactants [28,50,51], the charge transfer between water molecules [30,52] and the dipolar organization of interfacial water [31,53]. One of the most promising hypotheses, confirmed by our previous work [26] and experimental observations, is that the surface enrichment of charged particles, such as dissolved ionic surfactants and ions, from the medium really matters.

**Figure 4:**
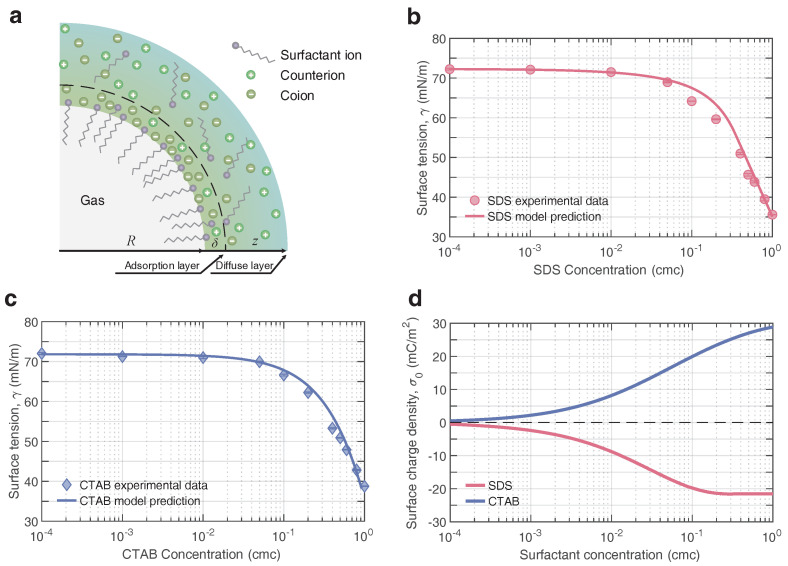
(**a**) Schematics of the adsorption layer structure of a negatively charged bulk nanobubble in anionic surfactant and electrolyte solutions. The sharp air–water interface is located at z=R mathematically. Surfactant molecules/ions and counterions around the surface form an adsorption layer of thickness δ, and the diffuse layer starts at z=R+δ. Calculated surface tension against experiment for (**b**) anionic surfactant (SDS) solution and (**c**) cationic surfactant (CTAB) solution. (**d**) Predicted surface charge density over a wide range of ionic surfactant concentrations.

When it comes to the distribution and adsorption of charged particles around the charged interface, the double-layer theory has been well used and can describe the ion distribution qualitatively. Figure 4 depicts the double layer structure of the bulk nanobubble in the aqueous solutions containing surfactants and electrolytes. To simplify the behavior of these charged particles in the diffuse layer in terms of the Boltzmann distribution, the majority of previous models merely take the electrostatic force into account. However, this simplification may cause some unexpected deviations in describing the distribution of surfactants and salts in the vicinity of the interfacial layer (adsorption layer), typically a few nanometers. Hence, the additional interactions between the charged particles and the interface should be seriously considered. In the experiments, we only used the monovalent ionic surfactant and electrolyte. For simplicity, the monovalent ionic surfactant and electrolyte were taken into consideration theoretically.

When a spherical charged nanobubble is suspended freely in an aqueous solution of dissolved surfactants and electrolytes, the distribution of species *i* near the bubble surface $c_{i}\left(z-R\right)$ can be obtained by the following Equation [54] with the center of the bubble as the origin of coordinates (see Figure 4a)

(1) $$c_{i}\left(z-R\right)=c_{0,i}\exp\left(-\frac{eq_{i}\psi \left(z-R\right)+\Delta W_{im}\left(z-R\right)+\Delta W_{it}\left(z-R\right)}{k_{B}T}\right),$$

where $c_{0,i}$ is the bulk concentration of species *i*, ψ(z−R) is the mean field potential as a function of distance z−R from the center of the bubble, $q_{i}$ denotes the valency of surfactant or ion type *i*, $\Delta W_{im}\left(z-R\right)$ is the image charge energy and $\Delta W_{it}\left(z-R\right)$ is the interaction energy between surfactants and interface. Here, all of the ions present in the liquid were assumed to be point charges, that is, they had no certain radius and polarizability. So the short-range interactions, such as ion-hydration force and cavitation potential, were excluded from this model. They were only subject to the dominant interaction, such as electrostatic force and image force [55,56]. The long-range interaction was added and described approximately via the image force $\Delta W_{im}\left(z-R\right)$. Moreover, a sharp air–water interface was considered (z−R>0), so that the volume concentration and permittivity of water molecules in the interfacial layer (adsorption layer) were consistent with those in the bulk phase [56,57,58]. Nevertheless, it should be noted that water molecules tend to have a more abnormal structure in the vicinity of the interface than in the bulk that may generate a repulsion on nearby ions. To date, this is still an open question, let alone incorporating its effect into the Poisson–Boltzmann formalism.

The mean field potential ψ(z−R) thus could be described by the modified Poisson–Boltzmann theory for z≥R

(2) $$\begin{array}{cc} \frac{d^{2}\psi \left(z-R\right)}{dz^{2}}= & -\frac{1}{\epsilon_{0}\epsilon_{w}}\sum_{i}ec_{0,i}q_{i}\exp\left(-\frac{eq_{i}\psi \left(z-R\right)+\Delta W_{im}\left(z-R\right)+\Delta W_{it}\left(z-R\right)}{k_{B}T}\right). \end{array}$$

Here, we only considered the surfactant ions and their counterions in bulk nanobubbles suspension owing to the fact that no extra electrolytes were introduced. As for $\Delta W_{im}\left(z-R\right)$, ignoring ion-specific effects, the Wagner form [59] was taken to describe the image charge repulsion from the gas–liquid interface

(3) $$\Delta W_{im}\left(z\right)=\frac{\epsilon_{w}-\epsilon_{a}}{\epsilon_{w}+\epsilon_{a}}\frac{e^{2}}{16\pi \epsilon_{0}\epsilon_{w}\left(z-R\right)}\exp\left(-\frac{2(z-R)}{\kappa^{-1}}\right)$$

where $\epsilon_{a}$ is the dielectric constant of air. $\kappa^{-1}$ is the Debye length $\kappa =\sqrt{(2N_{A}Ie^{2})/\left(\epsilon_{w}\epsilon_{0}k_{B}T\right)}$ [60]. Generally, the Debye length indicates the characteristic length-scale at which electrostatic screening interactions occur.

The $\Delta W_{it}\left(z-R\right)$ describes the interaction energy between the surfactant and the gas–liquid interface via a Gaussian form, which is represented by

(4) $$\Delta W_{it}\left(z-R\right)=A\exp\left[-{\left(\frac{z-R}{\delta }\right)}^{2}\right]$$

where *A* is the amplitude of interaction, and δ is the decay distance denoting the range of the interaction. It should be pointed out that depicting the interaction between surfactants and the gas–liquid interface is one great challenge. The dominating reason is that a realistic adsorption behavior of surfactant at the air–water interface has not yet been revealed. It is common to fit the parameters of the adsorption model on the basis of experimental surface tension measurements. Nevertheless, as has been reported [58,61], surface tension alone does not provide enough information to determine these two parameters simultaneously, and thus a fitting through experiments and simulations to calibrate is required. In reality, the parameters *A* and δ, substantially, depend on the species of surfactant and on characteristic parameters. In this work, $A_{1}=-15.2\;k_{B}T$ and δ=0.3c+1.5nm (where *c* is the bulk concentration of surfactant in mM) were taken for the anionic surfactant SDS [32,54,62]. Based on a similar adsorption characteristic, we set $A_{2}=-19.8\;k_{B}T$ and δ=0.3c+1.5nm for the cationic surfactant CTAB.

Once the complex interactions had been considered together, the potential ψ and concentration $c_{i}$ as a function of distance *z* could be obtained by solving the modified Poisson–Boltzmann equation numerically with the following boundary conditions

(5) $$\frac{d\psi (z-R)}{dz}{|}_{z-R=0}=0,\;\frac{d\psi (z-R)}{dz}{|}_{z-R=\infty }=0,$$

where the former represents a Gaussian theorem fitting feature at the interface, while the latter represents the charge neutrality in the bulk. Compared to a traditional adsorption model, this modified Poisson–Boltzmann model gave a more precise description of the diffuse layer and a more realistic adsorption layer with a finite thickness at the gas–liquid interface. Meanwhile, the distribution of surfactants and counterions as well as the potential adjacent to the interface were depicted on the basis of interaction. It should be noted that the explicit interaction between surfactants and counterions were not derived specifically due to the limitation of mean field approximation.

The difference between moles of the *i*th component at the interface and in the bulk was represented by surface excess $\Gamma_{i}$, which was given by

(6) $$\Gamma_{i}=\int_{R}^{+\infty }\left(c_{i}-c_{0,i}\right)dz.$$

According to the Gibbs convention, which means that the water appears to adopt bulk-like properties, here the Gibbs dividing surface was set at z=R. The total surface excess hence was defined by the difference between the surface excess of surfactants ($\Gamma_{1}$) and counterions ($\Gamma_{2}$), $\Gamma =\Gamma_{1}-\Gamma_{2}$. Furthermore, the quantitative relationship between the surface tension and surface excess is well understood by the classical Gibbs thermodynamics, which results in the Gibbs equation

(7) $$\frac{d\gamma }{k_{B}T}=\sum_{i}\Gamma_{i}d\ln c_{0,i}.$$

The surface tension thus could be calculated via Equations (1)–(7). Here, to verify the applicability and accuracy of the model, two ionic surfactants, anionic surfactant (SDS) and cationic surfactant (CTAB), were considered. Experimentally, we measured the air–liquid interfacial tension of surfactant solutions over a wide range of bulk concentrations. Figure 4b,c plot the calculated surface tension against experimental measurements for the SDS and CTAB solutions, respectively, indicating a very close agreement within four orders of magnitude between $10^{-4}$ and 1 cmc for both. Taking the adsorption feature of the surfactant and the adsorption layer thickness at the interface into consideration, this model was amenable and universal for the premicellar dilute solutions ($c_{0}\leq \mathrm{CMC}$).

Further, the surface charge density $\sigma_{0}$, which is a key physical quantity related to the stability of bulk nanobubbles, could be obtained based on the total surface excess Γ, essentially, the specific distribution of surfactant ions and counterions. Figure 4d shows how the calculated $\sigma_{0}$ in two ionic surfactant solutions depended on the bulk concentration. The magnitude of $\sigma_{0}$ rose steeply with the increasing concentration of the anionic surfactant SDS, especially when $c_{0}>10^{-3}$ cmc, and leveled off beyond 0.5 cmc, reaching a plateau value of around $-21.5\;\mathrm{mC}/m^{2}$. At high surfactant concentrations, the insensitivity of $\sigma_{0}$ to SDS concentration was attributed to the fact that the nanobubble surface had reached a saturated adsorption level, as stated earlier. This showed a similar trend to the surface excess at the gas–liquid interface in the SDS solution measured by neutron reflection [63] and radiotracer [64]. Generally, the surface charge density obtained with this model was consistent with the calculation based on Ohshima’s model [65] in previous work [25], which combined with the zeta potential measured in experiments. In contrast, this model quantitatively depicted the net accumulation of charges at the bulk nanobubble surface independent of the zeta potential. In contrast, the CTAB concentration dependence of the surface charge density exhibited a similar behavior except that there was no plateau at high concentrations, suggesting that the adsorption of CTAB molecules at the interface had not reached saturation [66].

### 3.3. Ion Distribution near the Gas–Liquid Interface

**Figure 5:**
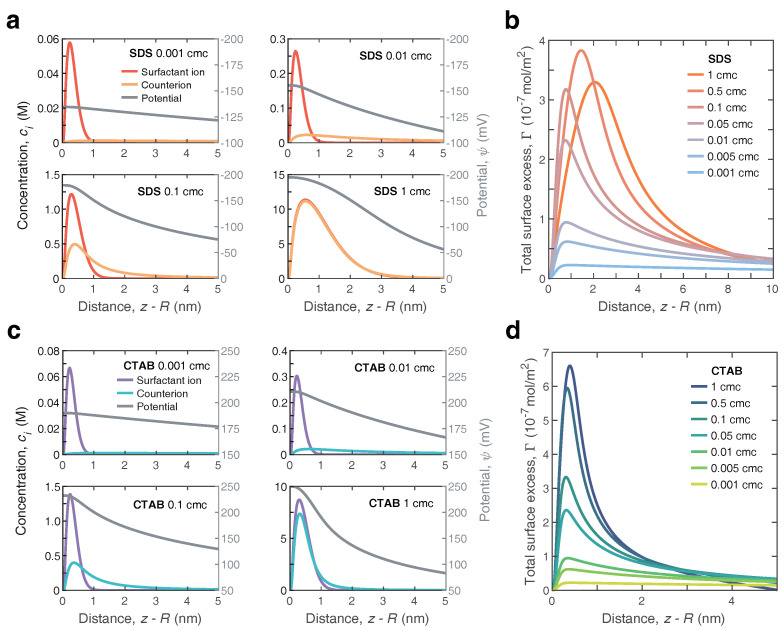
Distribution of anionic surfactant (SDS) and cationic surfactant (CTAB) in the vicinity of nanobubbles surface. (**a**) Concentration profile (left axis) of the surfactant ions (${\mathrm{DS}}^{-}$) and the counterions (${\mathrm{Na}}^{+}$), and the variation of the potential ψ (right axis) with distance z−R near the nanobubble interface for surfactant solutions with bulk concentration 0.001 cmc, 0.01 cmc, 0.1 cmc and 1 cmc. The critical micelle concentration of SDS is around 8.2 mM at 25 ${}^{{}^{\circ}}$C. (**b**) Spatial distribution of the total surface excess difference Γ near the nanobubble interface for the surfactant concentration ranging from 0.001 to 1 cmc. (**c**) Concentration profile (left axis) of the surfactant ions (${\mathrm{CTA}}^{+}$) and the counterions (${\mathrm{Br}}^{-}$), and the variation of the potential ψ (right axis) with distance z−R near the nanobubble interface for surfactant solutions with bulk concentration 0.001 cmc, 0.01 cmc, 0.1 cmc and 1 cmc. (**d**) Spatial distribution of the total surface excess difference Γ near the nanobubble interface for the surfactant concentration ranging from 0.001 to 1 cmc.

To gain a better insight into the underlying mechanism of adsorption, we now turn back to the details of the surface potential and ion distributions surrounding the nanobubbles. For the nanobubbles immersed in SDS solution, Figure 5a shows a series of computed spatial distribution of surfactant ions (${\mathrm{DS}}^{-}$) and counterions (${\mathrm{Na}}^{+}$) across the nanobubble interface (left *y*-axis). These four cases ($c_{m}$ = 0.001, 0.01, 0.1 and 1 cmc) were representative of all the cases considered. Generally, both the surfactant ions and counterions appeared to reach a plateau value very close to the interface (∼0.5nm), exhibiting an intense affinity for the interface. The concentrations of both decayed rapidly as they approached the sharp air–water interface indefinitely (<0.5nm). At a long enough distance (>∼3nm), these two profiles coalesced to a single decay. The decay distance varied from ∼1nm to ∼4nm with an increasing SDS concentration. However, surfactant ions (${\mathrm{DS}}^{-}$) displayed a greater propensity to gather near the air–water interface than counterions (${\mathrm{Na}}^{+}$) and tended to reside in the adsorption layer. At surfactant concentrations close to CMC, the counterions behaved similarly to the ${\mathrm{DS}}^{-}$ anions, and they overlapped extensively. Under this case, the surfactant layer was effectively screened by the counterion layer, even though the adsorption layer became denser. Nonetheless, the shape of the profiles within the thickness range of the adsorption layer was quite sensitive to the bulk concentration of ionic surfactants, essentially demonstrating that incorporating the effect of the adsorption thickness was crucial in the development of adsorbing dynamics. The corresponding electrical potential ψ as a function of radial distance near the nanobubble interface was also calculated and is shown in Figure 4a (right *y*-axis). Clearly, with an increasing SDS concentration, the ψ decayed faster away from the interface. Moreover, the shape of the potential profiles in the interface region behaved significantly distinctly from that obtained by the MPB model, i.e., as shown in our current work [26]. The potential predicted in this model was significantly higher than in the previous one owing to the difference in the definition of the surface. In Figure 5b, we further display the total surface excess Γ as a function of the distance away from the interface. Clearly, for all cases, the surfactant ions (${\mathrm{DS}}^{-}$) had a positive accumulation. As the bulk SDS concentration increased, the peak displayed a shift away from the air–water interface with a magnitude increasing remarkably.

As a comparison, in Figure 5c we show the spatial distribution of these same parameters surrounding the positively charged nanobubbles, which are suspended in CTAB solution. As apparent in Figure 5c, the spatial distribution of surfactant cations (${\mathrm{CTA}}^{+}$) could behave in a simpler manner, appearing to gather typically over a rather thin thickness of ∼1 nm around the interface and reaching a plateau value at z−R≈0.3nm. Moreover, the surfactant cations ${\mathrm{CTA}}^{+}$ dominated the adsorption layer at any bulk concentration, which eventually led to the net accumulation of positive charge, as shown in Figure 5d. The sharpening of the peak of total surface excess Γ with the increasing CTAB concentration indicated a more pronounced affinity of surfactant cations (${\mathrm{CTA}}^{+}$) than counterions (${\mathrm{Br}}^{-}$) for the interface.

**Figure 6:**
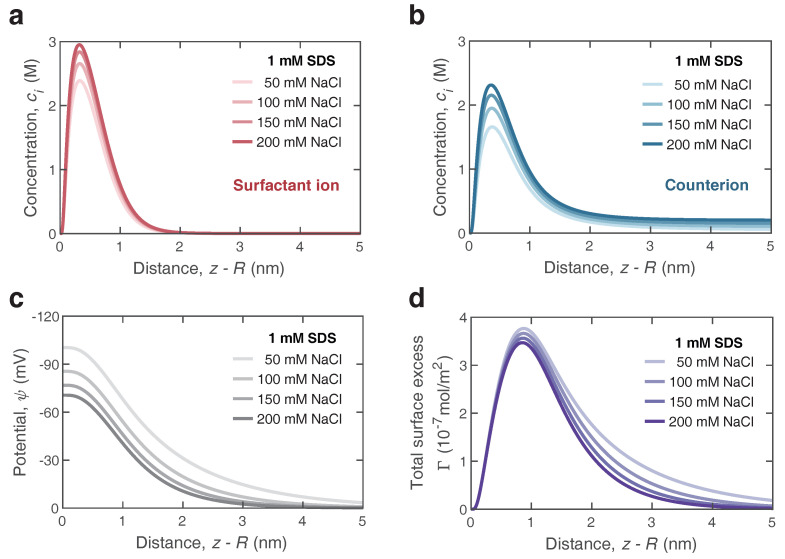
Coupling effect of anionic surfactant (SDS) and electrolyte (NaCl) on ion and potential distribution in the vicinity of nanobubble surface. (**a**,**b**) Concentration profile of the surfactant ions (${\mathrm{DS}}^{-}$) and the counterions (${\mathrm{Na}}^{+}$) with distance z−R near the surface of nanobubble in 1 mM SDS solutions mixed with NaCl at various concentrations. (**c**) Variation of the potential ψ as a function of distance. (**d**) Spatial distribution of the total surface excess difference Γ.

**Figure 7:**
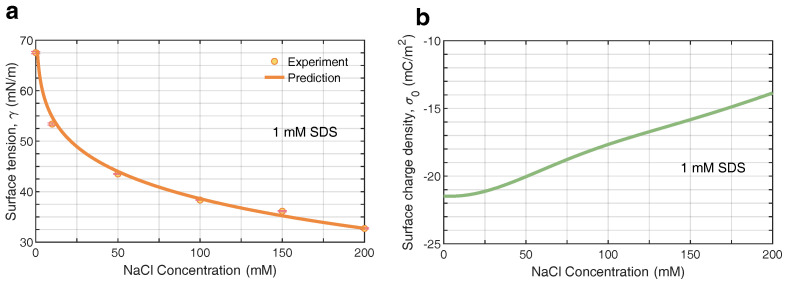
(**a**) Calculated surface tension against experiment for anionic surfactant (SDS) and electrolyte (NaCl) solutions at varying concentrations. The experimental data come from the reference [67]. (**b**) Predicted surface charge density of nanobubbles in surfactant and electrolyte solutions.

Further, we extended the adsorption model to describe the surface characteristics of bulk nanobubbles in the surfactant/electrolyte solutions. On the basis of the previous surfactant-counterion model, the influence on the mean field of potential arisen from the added electrolyte should be taken into consideration. For SDS/NaCl solutions, the parameters were $A_{3}=0.79\ln c-15.2k_{B}T$ (where *c* is the bulk concentration of salts in mM) and δ=1.8 nm. Figure 6a,b depict the profiles of surfactant ions and counterions in the vicinity of bulk nanobubbles, respectively, which were calculated with Equations (1) and (2). Here, we considered the distribution of surfactant ions and counterions in a solution with a constant bulk concentration of 1 mM SDS and a varying NaCl concentration ranging from 50 mM to 200 mM. The peaks of surfactants ions and counterions rose with the increasing concentration of electrolyte, indicating that the addition of electrolyte enhanced the adsorption of surfactant ions as well as counterions. It may be explained by the fact that the ions screened the electrostatic potential, allowing more surfactant molecules to adsorb to the surface of bulk nanobubbles. The preliminary conjecture was confirmed by the results of the electrical potential ψ in Figure 6c. The addition of NaCl reduced the electrical potential ψ at the air/water interface in the SDS solution significantly, due to the enhanced ion shielding effect. With an increasing distance away from the surface of bulk nanobubbles, the potential gradually dropped to zero within 5 nm due to the presence of NaCl in the SDS solution. The higher the concentration of NaCl, the faster the potential decayed. Since the adsorption was effective at a very close distance, the surfactant ions were concentrated near the interface and formed an adsorption layer. In contrast, the ion distribution in the diffuse layer was dominated by the electrostatic energy. According to the profiles of ion distribution, it could be found that more surfactant ions gathered at the interface with the addition of electrolytes in the surfactant solution, leading to a higher surface excess and a further lower surface tension. It was verified by the results of the surface tension predicted and measured in Figure 7a. Even with only 1 mM SDS, the surface tension of the aqueous solution could drop to 32 mN/m, which was lower than the surface tension of SDS near the CMC. Note that the concentration of 0 mM NaCl marks the SDS solution without any electrolytes added. Figure 7b shows the effect of NaCl on surface charge density. As the concentration of NaCl increased from 0 mM to 200 mM, the magnitude of bubble surface charge density declined to −14 $\mathrm{mC}/m^{2}$ gently. A similar trend with increasing concentration of NaCl was found in the experimental results of the zeta potential in Figure 2a. Overall, the addition of electrolytes to the surfactant solution easily made charged bubbles screened by counterions, which meant a reduction in colloidal stability.

**Figure 8:**
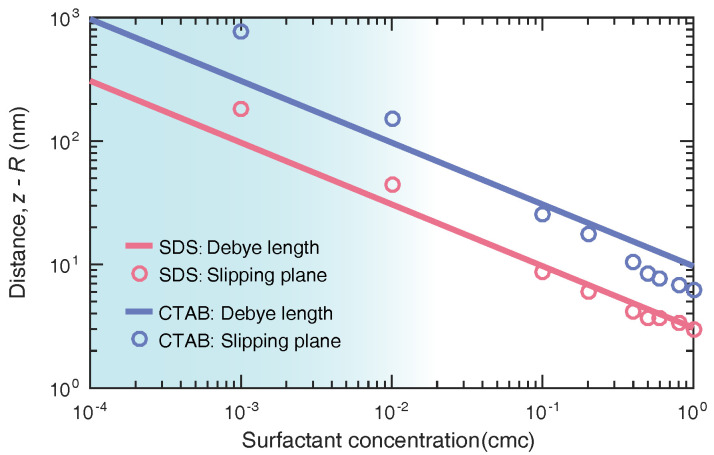
Prediction on the position of zeta potential measured in bulk nanobubble suspensions over a wide range of surfactant concentrations. The solid line denotes the corresponding Debye length. Note that the ionic surfactants were added to the pure water prior to the generation of bulk nanobubbles.

When the bubble is electrically neutral at a very close distance from the surface, it is easier to coalesce when moving randomly in solution. In other words, the spatial distribution of surfactant ions and counterions determines the position of the slipping plane. It is well-known that the zeta potential is the electrical potential at the slipping plane, which is a scientific term for colloidal electrokinetics. The zeta potential relies on the location of the slipping plane exceedingly. The accurate measuring plane around the nanobubble has been under controversy due to the lack of well-recognized depiction about the relation between potential and location. Here, we determined the slipping-plane location by substituting the measured zeta potential of bulk nanobubbles into the potential profile obtained with this model. Prior to ultrasonic treatment, the ionic surfactants were dispersed in the pure water. As shown in Figure 8, the slipping-plane positions of nanobubbles suspended in SDS and CTAB solutions were both almost located within the Debye layer (<$\kappa^{-1}$), which was in accordance with the assumption of the double layer theory. However, note that when the concentration of added surfactant was low, namely <$10^{-2}$ cmc (see the shaded area), the zeta potential was located beyond the Debye thickness. This discrepancy seems puzzling and may be traced to the fact that potential measurements are sensitive to charge carriers. If there are few charged carriers in the solution, the spatial distribution of the potential shows a slow decay far away the interface (as shown in Figure 5), which brings certain difficulties to accurately locate the experimental measurements. This issue needs to be further investigated by designing precise experiments, such as taking into account the coupling effect of ionic surfactants and coexisting ions, which is what we plan to do next.

Now going back to the charge-stabilization model, a single nanobubble has to obey the modified Young–Laplace equation, given that it is in a state of thermodynamic equilibrium. In short, the surface charge density $\sigma_{0}$ creates an electrostatic pressure $P_{e}=\sigma^{2}/2\epsilon_{w}\epsilon_{0}$, acting opposite to the Laplace pressure 2γ/R. When the surfactants and counterions attach onto the surface of bulk nanobubbles, the accumulation of net charges offer a repulsive electrostatic pressure while the reduced surface tension means a drop in the component of Laplace pressure, both of which contribute to the bulk nanobubbles’ thermodynamic stability. After calculation, $P_{e}$ was found to be of the same order of magnitude as the Laplace pressure contribution but it was still unsatisfactory to apply this model to quantitatively reproduce the experimental results. There is still a long way to go in rationalizing the stability of bulk nanobubbles.

## 4. Conclusions

In summary, we probed the coupling effect of surfactants and electrolytes on the nucleation and stability of bulk nanobubbles. The experimental results exhibited that bulk nanobubbles could nucleate and existed in the presence of the ionic surfactant and electrolyte. As the concentration of anionic surfactant increased, more nanobubbles were generated in solutions, featuring a narrower size contribution and a higher magnitude of zeta potential. It was conjectured that ionic surfactant molecules not only reduced the surface tension but also promoted the accumulation of net charges, which was beneficial to the nucleation and stabilization of bulk nanobubbles. The addition of electrolytes in surfactant solution resulted in a decrease in the zeta potential and concentration of nanobubbles due to the ion shielding effect; essentially, the colloidal stability of multiple nanobubbles suspended in surfactant solutions.

Further, we elucidated the adsorption of ionic surfactants and electrolyte at the gas–liquid interface; substantially, the accumulation of surface charges and the reduction of surface tension were responsible for the characteristics of bulk nanobubbles. Taking the adsorption layer thickness and interaction between surfactant ions and counterions into account, we developed a model for describing the surface potential and the distribution of surfactant ions and counterions around the nanobubble surface based on the modified Poisson–Boltzmann equation. The dependence of surface tension on bulk surfactant concentration was thus accurately captured. Our calculations revealed that the surface potential of bulk nanobubbles depended on both the adsorption layer thickness and the position of the slipping plane. The concentrations of surfactants and counterions near the nanobubble interface were generally several orders of magnitude higher than those in the bulk, showing a strong interfacial affinity. Moreover, the surface charge density predicted by this model was consistent with the analytical solution calculated by Ohshima’s model [25], which inherently depended on the experimentally measured zeta potential. This theoretical analysis has gone some way towards enhancing our understanding of the adsorption behavior of surfactants and ions in the vicinity of bubble surface.

Notwithstanding the effect of the surfactant and electrolyte on bulk nanobubbles explained using a charge adsorption model, there were also some assumptions that did not correspond to the real situation. Future work is under way to investigate the structuring of the gas–liquid interface of nanobubbles with molecular dynamics simulations, which can provide some insights to understand the thermodynamics of nanobubbles at the atomic scale. Our study will equip researchers and engineers in the field of bulk nanobubbles with a new perspective from which they can pay more attention to the properties of the gas–liquid interface and how the conundrum of thermodynamic equilibrium in bulk nanobubbles can be resolved going forward.

## Data Availability

Not applicable.

## References

[1] Tan B.H., An H., Ohl C.D. (2021). Stability of surface and bulk nanobubbles. Curr. Opin. Colloid Interface Sci..

[2] Zhang F., Sun L., Yang H., Gui X., Schönherr H., Kappl M., Cao Y., Xing Y. (2021). Recent advances for understanding the role of nanobubbles in particles flotation. Adv. Colloid Interface Sci..

[3] Zhu J., An H., Alheshibri M., Liu L., Terpstra P.M., Liu G., Craig V.S. (2016). Cleaning with bulk nanobubbles. Langmuir.

[4] Agarwal A., Ng W.J., Liu Y. (2011). Principle and applications of microbubble and nanobubble technology for water treatment. Chemosphere.

[5] Temesgen T., Bui T.T., Han M., Kim T.I., Park H. (2017). Micro and nanobubble technologies as a new horizon for water-treatment techniques: A review. Adv. Colloid Interface Sci..

[6] Gao Y., Li M., Sun C., Zhang X. (2022). Microbubble-enhanced water activation by cold plasma. Chem. Eng. J..

[7] Azevedo A., Oliveira H., Rubio J. (2019). Bulk nanobubbles in the mineral and environmental areas: Updating research and applications. Adv. Colloid Interface Sci..

[8] Ebina K., Shi K., Hirao M., Hashimoto J., Kawato Y., Kaneshiro S., Morimoto T., Koizumi K., Yoshikawa H. (2013). Oxygen and air nanobubble water solution promote the growth of plants, fishes, and mice. PLoS ONE.

[9] Yang H., Cai W., Xu L., Lv X., Qiao Y., Li P., Wu H., Yang Y., Zhang L., Duan Y. (2015). Nanobubble–Affibody: Novel ultrasound contrast agents for targeted molecular ultrasound imaging of tumor. Biomaterials.

[10] Wang Y., Li X., Zhou Y., Huang P., Xu Y. (2010). Preparation of nanobubbles for ultrasound imaging and intracelluar drug delivery. Int. J. Pharm..

[11] Eklund F., Alheshibri M., Swenson J. (2021). Differentiating bulk nanobubbles from nanodroplets and nanoparticles. Curr. Opin. Colloid Interface Sci..

[12] Epstein P.S., Plesset M.S. (1950). On the stability of gas bubbles in liquid-gas solutions. J. Chem. Phys..

[13] Alheshibri M., Qian J., Jehannin M., Craig V.S. (2016). A history of nanobubbles. Langmuir.

[14] Nirmalkar N., Pacek A., Barigou M. (2018). On the existence and stability of bulk nanobubbles. Langmuir.

[15] Ghaani M.R., Kusalik P.G., English N.J. (2020). Massive generation of metastable bulk nanobubbles in water by external electric fields. Sci. Adv..

[16] Taccoen N., Lequeux F., Gunes D.Z., Baroud C.N. (2016). Probing the mechanical strength of an armored bubble and its implication to particle-stabilized foams. Phys. Rev. X.

[17] Yasui K., Tuziuti T., Kanematsu W., Kato K. (2016). Dynamic equilibrium model for a bulk nanobubble and a microbubble partly covered with hydrophobic material. Langmuir.

[18] Zhang L., Chen H., Li Z., Fang H., Hu J. (2008). Long lifetime of nanobubbles due to high inner density. Sci. China Ser. G Phys. Mech. Astron..

[19] Attard P. (2014). The stability of nanobubbles. Eur. Phys. J. Spec. Top..

[20] Wang S., Zhou L., Wang X., Hu J., Li P., Lin G., Gao Y., Zhang L., Wang C. (2021). Collective dynamics of bulk nanobubbles with size-dependent surface tension. Langmuir.

[21] Li M., Ma X., Eisener J., Pfeiffer P., Ohl C.D., Sun C. (2021). How bulk nanobubbles are stable over a wide range of temperatures. J. Colloid Interface Sci..

[22] Nirmalkar N., Pacek A., Barigou M. (2018). Interpreting the interfacial and colloidal stability of bulk nanobubbles. Soft Matter.

[23] Meegoda J.N., Hewage S.A., Batagoda J.H. (2019). Application of the diffused double layer theory to nanobubbles. Langmuir.

[24] Tan B.H., An H., Ohl C.D. (2020). How bulk nanobubbles might survive. Phys. Rev. Lett..

[25] Zhang H., Guo Z., Zhang X. (2020). Surface enrichment of ions leads to the stability of bulk nanobubbles. Soft Matter.

[26] Ma X., Li M., Pfeiffer P., Eisener J., Ohl C.D., Sun C. (2022). Ion adsorption stabilizes bulk nanobubbles. J. Colloid Interface Sci..

[27] Peng M., Nguyen A.V. (2020). Adsorption of ionic surfactants at the air-water interface: The gap between theory and experiment. Adv. Colloid Interface Sci..

[28] Kim J.Y., Song M.G., Kim J.D. (2000). Zeta potential of nanobubbles generated by ultrasonication in aqueous alkyl polyglycoside solutions. J. Colloid Interface Sci..

[29] Gray-Weale A., Beattie J.K. (2009). An explanation for the charge on water’s surface. Phys. Chem. Chem. Phys..

[30] Vacha R., Marsalek O., Willard A.P., Bonthuis D.J., Netz R.R., Jungwirth P. (2012). Charge transfer between water molecules as the possible origin of the observed charging at the surface of pure water. J. Phys. Chem. Lett..

[31] Matyushov D.V. (2014). Electrophoretic mobility without charge driven by polarisation of the nanoparticle–water interface. Mol. Phys..

[32] Uematsu Y., Bonthuis D.J., Netz R.R. (2018). Charged surface-active impurities at nanomolar concentration induce Jones–Ray effect. J. Phys. Chem. Lett..

[33] Wang Q., Zhao H., Qi N., Qin Y., Zhang X., Li Y. (2019). Generation and stability of size-adjustable bulk nanobubbles based on periodic pressure change. Sci. Rep..

[34] Uchida T., Liu S., Enari M., Oshita S., Yamazaki K., Gohara K. (2016). Effect of NaCl on the lifetime of micro-and nanobubbles. Nanomaterials.

[35] Butt H.J., Graf K., Kappl M. (2013). Physics and Chemistry of Interfaces.

[36] Cho S.H., Kim J.Y., Chun J.H., Kim J.D. (2005). Ultrasonic formation of nanobubbles and their zeta-potentials in aqueous electrolyte and surfactant solutions. Colloids Surfaces A Physicochem. Eng. Asp..

[37] Parhizkar M., Edirisinghe M., Stride E. (2015). The effect of surfactant type and concentration on the size and stability of microbubbles produced in a capillary embedded T-junction device. Rsc Adv..

[38] Xu Q., Nakajima M., Ichikawa S., Nakamura N., Roy P., Okadome H., Shiina T. (2009). Effects of surfactant and electrolyte concentrations on bubble formation and stabilization. J. Colloid Interface Sci..

[39] Bui T.T., Nguyen D.C., Han M. (2019). Average size and zeta potential of nanobubbles in different reagent solutions. J. Nanopart. Res..

[40] Smoluchowski V., im unbegrenzten Raum I.D. (1906). Zusammenfassende bearbeitungen. Ann. Phys..

[41] Schindelin J., Arganda-Carreras I., Frise E., Kaynig V., Longair M., Pietzsch T., Preibisch S., Rueden C., Saalfeld S., Schmid B. (2012). Fiji: An open-source platform for biological-image analysis. Nat. Methods.

[42] Wagner T., Lipinski H.G., Wiemann M. (2014). Dark field nanoparticle tracking analysis for size characterization of plasmonic and non-plasmonic particles. J. Nanopart. Res..

[43] Takagi S., Matsumoto Y. (2011). Surfactant effects on bubble motion and bubbly flows. Annu. Rev. Fluid Mech..

[44] Craig V., Ninham B., Pashley R.M. (1993). Effect of electrolytes on bubble coalescence. Nature.

[45] Lee J.I., Kim J.M. (2022). Role of anionic surfactant in the generation of bulk nanobubbles by ultrasonication. Colloid Interface Sci. Commun..

[46] Nirmalkar N., Pacek A., Barigou M. (2019). Bulk nanobubbles from acoustically cavitated aqueous organic solvent mixtures. Langmuir.

[47] Jin F., Li J., Ye X., Wu C. (2007). Effects of pH and ionic strength on the stability of nanobubbles in aqueous solutions of *α*-cyclodextrin. J. Phys. Chem. B.

[48] Galloway W.J. (1954). An experimental study of acoustically induced cavitation in liquids. J. Acoust. Soc. Am..

[49] Khaled Abdella Ahmed A., Sun C., Hua L., Zhang Z., Zhang Y., Marhaba T., Zhang W. (2018). Colloidal properties of air, oxygen, and nitrogen nanobubbles in water: Effects of ionic strength, natural organic matters, and surfactants. Environ. Eng. Sci..

[50] Takahashi M. (2005). *ζ* potential of microbubbles in aqueous solutions: Electrical properties of the gas- water interface. J. Phys. Chem. B.

[51] Creux P., Lachaise J., Graciaa A., Beattie J.K. (2007). Specific cation effects at the hydroxide-charged air/water interface. J. Phys. Chem. C.

[52] Vácha R., Rick S.W., Jungwirth P., de Beer A.G., de Aguiar H.B., Samson J.S., Roke S. (2011). The orientation and charge of water at the hydrophobic oil droplet–water interface. J. Am. Chem. Soc..

[53] Smolentsev N., Roke S. (2020). Self-assembly at water nanodroplet interfaces quantified with nonlinear light scattering. Langmuir.

[54] Peng M., Duignan T.T., Zhao X.S., Nguyen A.V. (2020). Surface Potential Explained: A Surfactant Adsorption Model Incorporating Realistic Layer Thickness. J. Phys. Chem. B.

[55] Horinek D., Herz A., Vrbka L., Sedlmeier F., Mamatkulov S.I., Netz R.R. (2009). Specific ion adsorption at the air/water interface: The role of hydrophobic solvation. Chem. Phys. Lett..

[56] Manciu M., Ruckenstein E. (2012). Ions near the air/water interface: I. Compatibility of zeta potential and surface tension experiments. Colloids Surfaces A Physicochem. Eng. Asp..

[57] Karraker K., Radke C. (2002). Disjoining pressures, zeta potentials and surface tensions of aqueous non-ionic surfactant/electrolyte solutions: Theory and comparison to experiment. Adv. Colloid Interface Sci..

[58] Duignan T.T., Peng M., Nguyen A.V., Zhao X., Baer M.D., Mundy C.J. (2018). Detecting the undetectable: The role of trace surfactant in the Jones-Ray effect. J. Chem. Phys..

[59] Manciu M., Ruckenstein E. (2003). Specific ion effects via ion hydration: I. Surface tension. Adv. Colloid Interface Sci..

[60] Israelachvili J.N. (2011). Intermolecular and Surface Forces.

[61] dos Santos A.P., Levin Y. (2010). Surface tensions and surface potentials of acid solutions. J. Chem. Phys..

[62] Lu J., Thomas R., Penfold J. (2000). Surfactant layers at the air/water interface: Structure and composition. Adv. Colloid Interface Sci..

[63] Xu H., Li P.X., Ma K., Thomas R.K., Penfold J., Lu J.R. (2013). Limitations in the application of the Gibbs equation to anionic surfactants at the air/water surface: Sodium dodecylsulfate and sodium dodecylmonooxyethylenesulfate above and below the CMC. Langmuir.

[64] Tajima K., Muramatsu M., Sasaki T. (1970). Radiotracer studies on adsorption of surface active substance at aqueous surface. I. Accurate measurement of adsorption of tritiated sodium dodecylsulfate. Bull. Chem. Soc. Jpn..

[65] Ohshima H., Healy T.W., White L.R. (1982). Accurate analytic expressions for the surface charge density/surface potential relationship and double-layer potential distribution for a spherical colloidal particle. J. Colloid Interface Sci..

[66] Wang X., Santo K.P., Neimark A.V. (2020). Modeling gas–liquid interfaces by dissipative particle dynamics: Adsorption and surface tension of cetyl trimethyl ammonium bromide at the air–water interface. Langmuir.

[67] Peng M., Duignan T.T., Nguyen A.V. (2020). Quantifying the Counterion-Specific Effect on Surfactant Adsorption Using Modeling, Simulation, and Experiments. Langmuir.

